# PVAT and Its Relation to Brown, Beige, and White Adipose Tissue in Development and Function

**DOI:** 10.3389/fphys.2018.00070

**Published:** 2018-02-06

**Authors:** Staffan Hildebrand, Jasmin Stümer, Alexander Pfeifer

**Affiliations:** Institute of Pharmacology and Toxicology, University Hospital Bonn, University of Bonn, Bonn, Germany

**Keywords:** PVAT, perivascular, adipose tissue, BAT, inflammation, cardiovascular disease

## Abstract

Adipose tissue is commonly categorized into three types with distinct functions, phenotypes, and anatomical localizations. White adipose tissue (WAT) is the major energy store; the largest depots of WAT are found in subcutaneous or intravisceral sites. Brown adipose tissue (BAT) is responsible for energy dissipation during cold-exposure (i.e., non-shivering thermogenesis) and is primarily located in the interscapular region. Beige or brite (brown-in-white) adipose tissue can be found interspersed in WAT and can attain a brown-like phenotype. These three types of tissues also have endocrine functions and play major roles in whole body metabolism especially in obesity and its co-morbidities, such as cardiovascular disease. Over the last years, perivascular adipose tissue (PVAT) has emerged as an adipose organ with endocrine and paracrine functions. Pro and anti-inflammatory agents released by PVAT affect vascular health, and are implicated in the inflammatory aspects of atherosclerosis. PVAT shares several of the defining characteristics of brown adipose tissue, including its cellular morphology and expression of thermogenic genes characteristic for brown adipocytes. However, PVATs from different vessels are phenotypically different, and significant developmental differences exist between PVAT and other adipose tissues. Whether PVAT represents classical BAT, beige adipose tissue, or WAT with changing characteristics, is unclear. In this review, we summarize the current knowledge on how PVAT relates to other types of adipose tissue, both in terms of functionality, developmental origins, and its role in obesity-related cardiovascular disease and inflammation.

## Introduction

During the last decades, the prevalence of obesity has reached pandemic dimensions, doubling since 1990. In 2015, over 600 million obese adults and over 100 million obese children were reported worldwide (Afshin et al., 2017). Obesity is characterized by a high body mass index (BMI) ≥30. Overweight and obesity are associated with several severe comorbidities, such as cardiovascular disease, type 2 diabetes mellitus, and certain types of cancer (Chen et al., 2016). Importantly, more than two thirds of the deaths related to overweight and obesity were caused by cardiovascular disease (CVD) (Afshin et al., 2017) showing the importance of vascular disease in metabolic disorders. The defining trait in obesity is the abnormal increase in WAT mass with adipocyte hypertrophy and hyperplasia, brought on by an imbalance between energy intake and energy consumption leading to energy overload. This can result in hypertriglyceridemia, insulin resistance, and chronic low-grade inflammation first of the adipose tissue and then throughout the whole body (Czech et al., 1977; Hotamisligil, 2006; Guilherme et al., 2008).

**Figure 1:**
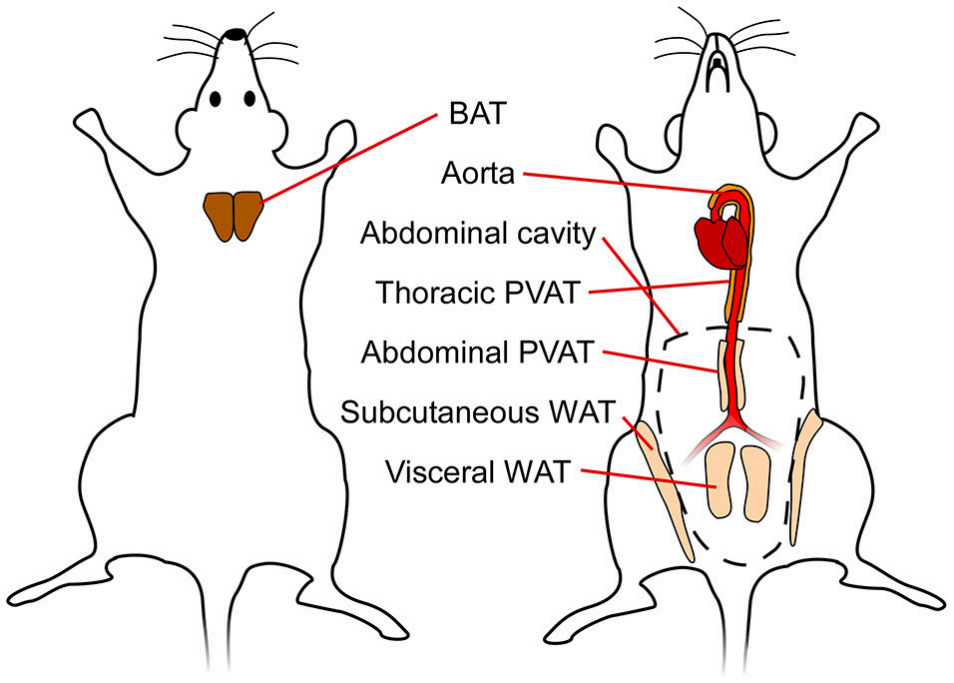
Simplified schematic showing different adipose tissue depots in mice. BAT, brown adipose tissue; WAT, white adipose tissue; PVAT, perivascular adipose tissue.

In mammals, there are three types of adipose tissues: white, brown and beige. These tissues have distinct functions and consequently have different morphology, protein expression patterns, and developmental origin (Pfeifer and Hoffmann, 2015). The function of white adipose tissue (WAT) is to store energy in the form of lipids, which can be released to fuel other tissues. On the other hand, brown adipose tissue (BAT) has unique thermogenic properties and is a vital organ for maintaining body temperature in smaller mammals and human infants with a high surface-to-volume ratio. Beige or brite (brown-in-white) fat is predominantly found interspersed in WAT depots, but can acquire a brown-like phenotype upon cold exposure or pharmacological stimulation (Chen et al., 2016). In mice, subcutaneous WAT (SAT) has the highest capacity for “browning” or “beiging” (Chen et al., 2016). Figure 1 shows a simplified schematic of the different (murine) adipose tissues discussed in this review.

**Figure 2:**
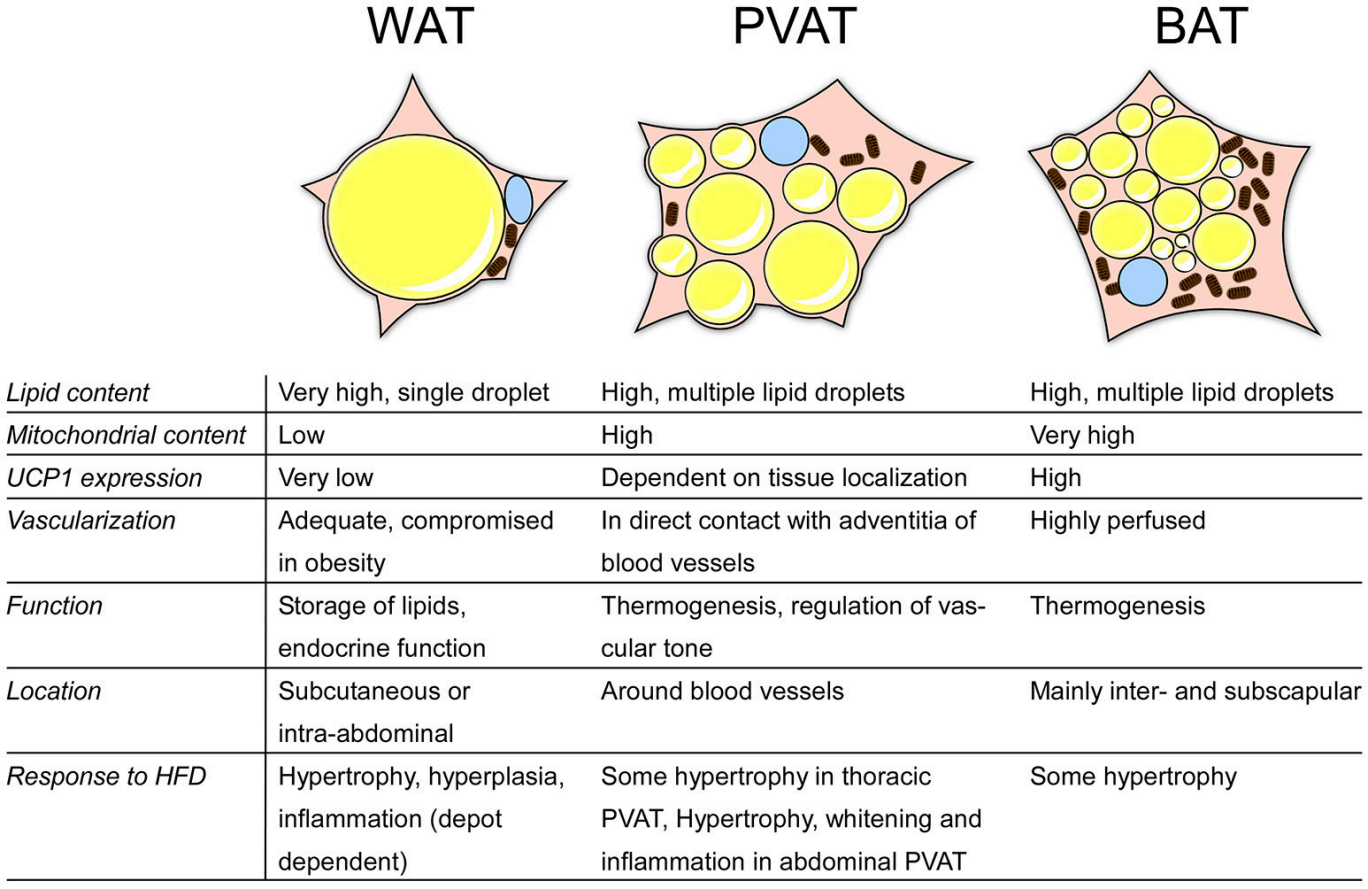
Overview of the characteristics of different adipose tissues. BAT, brown adipose tissue; WAT, white adipose tisse; PVAT, perivascular adipose tissue; HFD, high-fat diet; UCP1, uncoupling protein 1.

Perivascular adipose tissue (PVAT) is the fat surrounding the blood vessels, directly adjacent to the vascular wall, and was considered for long time to serve only structural, vessel-supporting purposes. As fat in general is now well known to be a secretory organ, PVAT is today also recognized to be an endocrine organ, actively releasing bioactive molecules such as pro- and anti-inflammatory cytokines and vasoactive substances (Figure 2; Soltis and Cassis, 1991; Gollasch and Dubrovska, 2004; Gao et al., 2007; Britton and Fox, 2011). Over the last years, several studies have been conducted comparing PVAT to other adipose tissues, such as classical BAT and WAT, which we try to summarize in this review. Obesity is associated with an increased risk for CVD and with remodeling of the adipose tissues, both WAT and BAT (Fantuzzi and Mazzone, 2007; Berbée et al., 2015). Given the proximity of PVAT to the vasculature, we also aim to compare the potential impact of PVAT on CVD and vice-versa.

### White adipose tissue

WAT makes up the main mass of adipose tissue in human adults, and represents 10-20 percent of body weight in healthy subjects. WAT is widely distributed through the whole body and is located mainly in subcutaneous regions and surrounding internal organs (visceral adipose tissue, VAT) (Chen et al., 2017). WAT is well known as the major organ to store energy in form of triacylglycerols (TAG), which can be mobilized via lipolysis whenever energy is needed. Lipolysis is initiated by norepinephrine binding to beta-adrenergic receptors. This initiates the production of cyclic adenosine-monophosphate (cAMP), the second messenger which activates hormone sensitive lipase via protein kinase A (PKA), resulting in the release of free fatty acids from stored TAG (Duncan et al., 2007).

Morphologically, white adipocytes contain a single, big lipid droplet occupying most of the cytoplasm, and a peripheral nucleus, which leads to a typical signet ring appearance. WAT also has important endocrine functions secreting hormones and cytokines such as leptin, adiponectin, tumor-necrosis factor α (TNFα) and interleukin-6 (IL-6) (Chen et al., 2017). Adipogenic differentiation of white adipocytes is regulated by several known transcription factors including CCAAT/enhancer-binding-proteins C/EBPβ and C/EBPδ, which in turn regulate expression of peroxisome proliferator-activated factor gamma (PPARγ) and C/EBPα. Together, C/EBPα and PPARγ regulate gene transcription and promote differentiation of adipocytes during late WAT adipogenesis (Barak et al., 1999; Rosen et al., 1999; Rosen and MacDougald, 2006; Hudak and Sul, 2013).

### Brown adipose tissue

BAT plays a crucial role in generating heat via non-shivering thermogenesis (NST) in newborn humans. NST is achieved through the expression of the mitochondrial protein uncoupling protein-1 (UCP-1), which uncouples the respiratory chain and causes a leak of protons across the mitochondrial membrane (Cannon and Nedergaard, 2004; Pfeifer and Hoffmann, 2015). This process results in the generation of heat instead of adenosine triphosphate (ATP), and is initiated by activation of β-3 adrenergic receptors (β3-AR) and adenosine A_2A_ receptors expressed on brown adipocytes (Lowell and Flier, 1997; Gnad et al., 2014). In contrast to white adipocytes, brown adipocytes contain many small lipid droplets leading to a multilocular histological appearance. Furthermore, brown adipocytes contain densely packed mitochondria needed for efficient NST, and is highly vascularised, which taken together causes the characteristic brown color.

Several factors that regulate BAT development have been identified, such as PPARγ, peroxisome proliferator-activated receptor gamma coactivator 1-alpha (PGC-1α), orexin, and bone morphogenic factor 7 (BMP7) (Tseng et al., 2008; Hondares et al., 2011; Cohen et al., 2014).

Nowadays, it is well established that not only newborns but also human adults have depots of BAT that are metabolically active during cold exposure. BAT in human adults is mainly found in the supraclavicular, neck, perirenal and mediastinal region (Nedergaard et al., 2007; Cypess et al., 2009; van Marken Lichtenbelt et al., 2009). These findings are is also in agreement with earlier post mortem investigations of human adults tissue showing that BAT can be found in deeper regions of the human body, where it might act as a thermogenic protection for internal organs (Heaton, 1972). Interestingly, studies in human adults have demonstrated a reduced BAT activity in obese and overweight subjects. Conversely, BAT mass positively correlates with resting metabolic rate (van Marken Lichtenbelt et al., 2009). During obesity, the adipocytes in interscapular BAT seem to adopt a white-like phenotype, with increased lipid accumulation (Shimizu et al., 2014).

In summary, the presence of active BAT in human adults makes this special type of fat an interesting target for new therapeutic approaches to tackle obesity.

### Beige adipose tissue

In response to cold exposure, WAT can adopt a brown-like phenotype in a process called “browning.” During browning, UCP-1-expressing brown-like adipocytes, with a high number of mitochondria and multilocular lipid droplets, appear (Lo and Sun, 2013; Pfeifer and Hoffmann, 2015; Chen et al., 2016). These so called beige or brite (brown-in-white) cells also express a number of characteristic markers, such as CD137, Tbx1, and Cited-1 (Harms and Seale, 2013). The capacity for browning varies between the different WAT depots, with SAT being more prone to browning than VAT (Seale et al., 2011). This probably owes mainly to differential expression of PR domain containing 16 (PRDM16) in SAT and VAT. PRDM16 is critical for phenotypic maintenance of classical BAT, and is more highly expressed in SAT than VAT (Seale et al., 2011). Importantly, ablation of PRDM16 in adipocytes disrupts browning of SAT upon cold exposure (Cohen et al., 2014). The interconversion of adipocytes has been reported to be possible in both directions: white adipocytes gain a beige/brite phenotype during cold exposure, and return to a white adipocyte-like appearance after removal of the cold stimulus (Rosenwald et al., 2013).

## PVAT as brown adipose tissue

### Similarities and differences between PVAT vs. BAT, WAT, or beige fat

Several reports (Gálvez-Prieto et al., 2008; Police et al., 2009; Fitzgibbons et al., 2011) indicate that depending on the localization, PVAT can resemble either WAT or BAT. Thoracic periaortic adipose tissue is morphologically similar to BAT, with adipocytes that have a multilocular appearance and round nuclei (Fitzgibbons et al., 2011). A direct comparison of gene expression of thoracic PVAT, interscapular BAT and WAT from mice revealed that only 228 genes (i.e. 0.79%) were significantly different between thoracic PVAT and classical BAT (Fitzgibbons et al., 2011). Interestingly, there was no significant difference in the expression levels of the genes known to be typically expressed in classical BAT, such as *Cidea, Ucp-1*, or *PPAR*γ (Fitzgibbons et al., 2011). Furthermore, proteomic analysis shows striking similarities in protein expression between periaortic adipose tissue and classical BAT, but not WAT (Chang et al., 2012).

In contrast to beige adipose tissue, thoracic PVAT maintains a BAT-like phenotype in the absence of activating stimuli. Furthermore, perivascular adipocytes do not undergo significant whitening during high fat diet (HFD) feeding, which is characteristic of BAT (Fitzgibbons et al., 2011). These findings suggest that, from a morphological and functional standpoint, thoracic PVAT more closely resembles classical BAT than beige fat.

Functionally, PVAT also exhibits important similarities with BAT. Chang et al. created a PVAT-deficient mouse by knocking out PPARγ in smooth muscle cells, and were able to show that PVAT significantly contributes to maintaining intravascular temperature during cold exposure. While PVAT activation could not completely rescue the reduction in intravascular temperature caused by resection of BAT, mice lacking both BAT and PVAT had significantly lower intravascular temperature than mice lacking only BAT (Chang et al., 2012).

However, there are also reports characterizing PVAT as WAT or WAT-like (Omar et al., 2014). This discrepancy appears to derive from the anatomical localization of the PVAT. In contrast to thoracic PVAT, the adipose tissue surrounding the abdominal aorta appears to be similar to WAT. In obese mice, the abdominal PVAT has been described to have a white-like phenotype with a primarily unilocular appearance (Police et al., 2009). Additionally, the mesenteric PVAT has been described to be similar to WAT with large lipid droplets and low expression levels of UCP-1 (Gálvez-Prieto et al., 2008).

Together, these data suggest that the functional phenotype of PVAT is directly linked with its anatomical localization. Thoracic PVAT is phenotypically close to BAT, and shares its unique functional characteristics. Abdominal PVAT, on the other hand, has been described to closely resemble WAT, and has a similar role in obesity-induced inflammatory responses. Furthermore, PVAT does not seem to share the dynamic properties of beige fat: thoracic PVAT displays a BAT-like phenotype without external stimuli, and does not readily lose this phenotype in diet-induced obesity.

### Developmental differences between PVAT, BAT, WAT, and beige fat

While the phenotype of thoracic PVAT may be almost indistinguishable from BAT, the question whether or not it is classical BAT is still not solved. On the other hand, location of PVAT might determine its color/phenotype: some parts of PVAT, e.g., abdominal PVAT, have been reported to resemble WAT (Police et al., 2009; Gálvez-Prieto et al., 2012). During embryogenesis, the mesodermal germ layer gives rise to mesenchymal progenitors that in turn differentiate into all types of adipocyte precursor cells. However, over the last decade, it has become increasingly clear that adipocytes of different adipose tissue depots are derived from precursor cells of distinct lineages. This notion is underlined by the fact that mature adipocytes of different depots appear at different times during embryogenesis (Xue et al., 2007; Wang et al., 2013; Hong et al., 2015). Functional mature brown adipocytes in the interscapular region are necessary for temperature homeostasis immediately at birth, and consequently start developing around 4 days prior to birth (Xue et al., 2007). White adipocytes mostly develop after birth, although significant differences exist between WAT tissues in different depots. This was recently demonstrated by Wang et al., who used an inducible adipocyte labeling system based on the adiponectin promoter to track adipogenesis *in vivo* (Wang et al., 2013). In this study, visceral adipocytes were found to start developing postnatally, while subcutaneous adipocytes initiated differentiation in the embryo (around E16) (Wang et al., 2013). The latter finding is also corroborated by another study, where flow cytometry and histological analysis revealed a subcutaneous population of lipid-lacking perilipin^+^/adiponectin^+^ preadipocytes appearing at E16.5 (Hong et al., 2015). Considering the phenotypical differences between PVAT surrounding different vessels, it is plausible that location-specific differences in PVAT development also exist.

Aside from the temporal regulation of adipocyte differentiation, differences can also be found in the progenitor cells themselves that give rise to mature adipocytes of different depots. *De novo* adipogenesis of white fat occurs close to blood vessels, and several studies have demonstrated that white adipocytes develop from perivascular platelet-derived growth factor α-expressing (Pdgfrα^+^) progenitor cells (Berry and Rodeheffer, 2013; Hong et al., 2015; Sun et al., 2017). However, adipocyte progenitors cannot be identified solely based on Pdgfrα expression, as not all vascular Pdgfrα^+^ cells are adipogenic (Berry and Rodeheffer, 2013). Other studies have shown that the vascular fraction capable of *in vitro* adipogenesis is CD31^−^CD34^+^, which is an antigen signature that matches adventitial fibroblasts rather than endothelial or mural cells (Guimaraes-Camboa and Evans, 2017; Hepler and Gupta, 2017). White adipocytes that develop in adulthood, e.g., during obesity-induced WAT hyperplasia, likely have a different origin. Here, mature adipocytes derive from specialized mural Pdgfrβ^+^ precursor cells residing in the blood vessels of adipose tissue, although the exact identity of these cells is unknown (Jiang et al., 2014; Vishvanath et al., 2016).

On the other hand, brown adipocytes appear to stem from myogenic progenitors, and indeed share many characteristics with skeletal muscle cells, such as a similar transcriptome and mitochondrial proteome (Forner et al., 2009). The myogenic transcription factors paired box protein Pax-3 and 7 (Pax3, Pax7), as well as myogenic factor 5 (Myf5), are activated during the early development of brown adipocytes from mesenchymal stem cells (Lepper and Fan, 2010; Sanchez-Gurmaches and Guertin, 2014). From these Myf5^+^Pax3^+^Pax7^+^ precursors, brown preadipocytes become committed to the brown fat lineage through activation of BMP7 (Park et al., 2013). An earlier study identified PRDM16 as a key determinant of brown adipocyte commitment during development (Seale et al., 2008), but more recent investigations suggest that PRDM16 primarily maintains the BAT phenotype postnatally (Harms et al., 2014). The myogenic lineage described above may not be accurate for all BAT depots, as a recent study, in which detailed lineage analysis were performed, revealed that only the major depots (inter- and subscapular) of BAT are exclusively derived from Myf5^+^Pax3^+^ precursors. Furthermore, the myogenic lineage may also not be unique to BAT (Sanchez-Gurmaches and Guertin, 2014): some WAT depots are in fact derived solely from Myf5^+^ cells (Sanchez-Gurmaches and Guertin, 2014). This suggests that certain canonical BAT lineage markers may correlate more closely to anatomical localization of the tissue during development, rather than functionality.

Beige cells are often described as inducible brown adipocytes, although there is no consensus concerning the embryonic origin of beige adipocytes (Pfeifer and Hoffmann, 2015). Four possible lineages of beige adipocyte development have been suggested: (1) transdifferentiation of mature white adipocytes (Himms-Hagen et al., 2000; Vitali et al., 2012), (2) maturation of brown preadipocytes already existing in WAT (Wang et al., 2014), (3) differentiation and maturation of pre-existing white preadipocytes (Seale et al., 2008), or (4) differentiation from vascular precursors, similarly to what occurs during WAT hyperplasia (Long et al., 2014). In fact, the amounting data from recent studies suggest that all these pathways could contribute to beige adipocyte development, depending on tissue depot and stimuli (Harms and Seale, 2013). While transdifferentiation of mature adipocytes probably only takes place on a low scale (Harms and Seale, 2013), beige adipocytes can arise from brown-like preadipocytes (Myf5^+^) or white-like preadipocytes (Myf5^−^) depending on the developmental origin of the depot in question (Sanchez-Gurmaches et al., 2012). However, it is not presently clear whether this diverging lineage translates into functional differences. Furthermore, prolonged cold exposure in rodents (>2 weeks) leads to beige adipogenesis from the same mural Pdgfrβ^+^ precursor population that is responsible for diet-induced WAT hyperplasia (Vishvanath et al., 2016).

Perivascular fat does not share the myogenic lineage of classical brown fat. Rather, PVAT seems to share a developmental origin with vascular smooth muscle (mural) cells. Chang and colleagues deleted the master regulator of adipogenesis PPARγ in smooth muscle by crossing SM22α-Cre mice with PPARγ^flox/flox^ mice (Chang et al., 2012). This resulted in a complete loss of PVAT, but no change in either WAT or BAT development. The expression of SM22α in mesenchymal cells proximal to the aorta during early embryogenesis might suggest that PVAT development is initiated during embryonic development, which would distinguish it from other mural-derived adipose tissues (Li et al., 1996). However, further investigation is needed to clarify this.

Additionally, thoracic PVAT cells originate from Myf5^−^ precursors, further separating them from intrascapular classical brown adipocytes (Sanchez-Gurmaches and Guertin, 2014). Thoracic PVAT also seems to develop differently in males and females: a majority of periaortic adipocytes in females originate from Pax3^+^ precursors, while all periaortic adipocytes in males arise from Pax3^−^ cells. In contrast, 99–100% of all interscapular and subscapular brown adipocytes from both genders are derived from Pax3^+^ precursors (Sanchez-Gurmaches and Guertin, 2014). In summary, these data suggest that the functional differences between thoracic PVAT and BAT are almost negligible. In contrast, abdominal PVAT has been suggested to closely resemble WAT (Police et al., 2009; Gálvez-Prieto et al., 2012). PVAT might be categorized as a fourth type of fat tissue which seems to be developmentally different from BAT, WAT, and beige fat.

## PVAT in obesity-related vascular disease

### Atherosclerosis

CVD is the leading cause of mortality globally, and is responsible for almost a third of all deaths world-wide (WHO, 2017). The underlying cause of CVD is atherosclerosis, the thickening and hardening of arterial walls because of: (1) endothelial dysfunction, (2) retention and accumulation of low-density lipoprotein (LDL) particles and immune cells in the tunica intima, and (3) proliferation and migration of intimal smooth muscle cells. If unaddressed, this condition can progress to various life-threatening conditions such as thrombosis, myocardial infarction, and stroke (Lusis, 2000).

Inflammation is intimately linked with atherosclerosis. The initial step in atherogenesis is endothelial dysfunction, leading to increased retention of LDL particles in the subendothelial space. The retained particles are then modified in various ways (e.g., oxidation and glycation), which turns them into auto-antigens, inducing a low-grade inflammation (Rader and Daugherty, 2008; Tabas et al., 2015). The inflammatory response activates endothelial cells, leading to increased adhesion and infiltration and monocytes (Madamanchi et al., 2005; Tabas et al., 2015). Intra-intimal differentiation of monocytes into macrophages plays a key role in atherogenesis, and accumulation of the modified LDL particles in these macrophages eventually turns them into the foam cells that are characteristic of atherosclerosis (Tabas et al., 2015). The adaptive immune system is also important for atherogenesis. T-cells and B-cells infiltrate the intima following endothelial cell activation, and have both been demonstrated to regulate the progression of atherosclerosis (Ammirati et al., 2015). Immunodeficiency in mice reduces plaque formation, and reconstituting CD4^+^ T-cells in *scid/scid* mice increases it (Dansky et al., 1997; Zhou et al., 2000). Furthermore, selective depletion of CD4^+^ T-cells using antibodies reduces fatty streak formation in early atherogenesis (Emeson et al., 1996). On the other hand, B-cell depletion by splenectomy increases atherogenesis, and subsequent B-cell transfer from donor mice decreases it, possibly through regulation of T-cell activity (Caligiuri et al., 2002).

### PVAT and inflammation

While conventional monocyte and T-cell infiltration occurs from the luminal side of the vessel wall, accumulating evidence also suggests that adventitia plays an important role in vascular inflammation. T-cells, B-cells, monocytes, and mature macrophages all reside in the adventitia of diseased vessels (Maiellaro and Taylor, 2007), the former two exceeding their corresponding numbers in the intima of ApoE^−/−^ mice up to 80-fold. Furthermore, adventitial vasa vasorum neovascularisation has been shown to precede endothelial dysfunction in hypercholesterolemic pigs (Herrmann et al., 2001). This suggests that immune cell infiltration occurs not only from the luminal side of the vessel (inside-out), but also from the adventitial side (outside-in) (Kawabe and Hasebe, 2014). Considering this, the role of PVAT in arterial inflammation is of great interest due to its location to the vessel wall, especially considering the lack of a fascia separating the PVAT from the adventitia. This direct contact enables significant paracrine signaling from PVAT to the vessel wall, and PVAT is indeed known to release numerous paracrine factors that influence the vessel in terms of both inflammation and contractility. Pro- and anti-inflammatory agents released by PVAT include leptin (Gálvez-Prieto et al., 2012; Li et al., 2014), adiponectin (Lynch et al., 2013; Antonopoulos et al., 2015), resistin (Park et al., 2014), TNF-α (DeVallance et al., 2016), MCP-1 (Manka et al., 2014), TGF-β (Chatterjee et al., 2013), angiopoietin-like protein 2 (Angptl2) (Tian et al., 2013), and IL-6 (Du et al., 2015), all of which are known to influence the progression of atherosclerosis in some way:

Adenoviral overexpression of leptin in PVAT promotes neointima formation, and transplantation of PVAT from HFD-fed obese mice, but not leptin-deficient *ob/ob* mice, to injured vessels increases neointima formation (Schroeter et al., 2013). Additionally, vascular smooth muscle cells (VSMCs) incubated with conditioned medium from the PVAT of HFD-fed rats increased leptin-dependent switching to the proliferative, synthetic phenotype characteristic of VSMCs in the neointima (Li et al., 2014).

Adiponectin is widely described as anti-inflammatory and protective against atherosclerosis (Xita and Tsatsoulis, 2012; Antonopoulos et al., 2015). Adiponectin-deficient mice have pronounced neointima formation upon wire injury, which can be rescued by local administration of recombinant adiponectin to the adventitial region of the injured vessels (Takaoka et al., 2009). This suggests that adiponectin secreted from PVAT may be protective against neointimal hyperplasia.

PVAT-derived resistin has not been directly shown to influence atherosclerosis, but does increase the expression of osteopontin in VSMCs (Park et al., 2014), which in turn has been implicated in VSMC proliferation and restenosis (Panda et al., 1997; Shimizu et al., 2004).

The effect of PVAT on intimal VSMC infiltration has also been investigated after transplantation of thoracic PVAT to wire-injured carotid arteries, where the presence of PVAT accelerated neointimal formation in an MCP-1 dependent manner (Manka et al., 2014). Interestingly, MCP-1 did not influence the infiltration of macrophages. MCP-1 has also been shown to stimulate VSMC proliferation *in vitro* (Viedt et al., 2002).

Moreover, inflamed PVAT increases VSMC proliferation in a TGF-β dependent manner, suggesting that TGF-β released from PVAT can potentiate neointima formation (Moe et al., 2013) The PVAT transplantation approach has also been used to study the effects of PVAT-derived Angptl2 on the progression of neointimal hyperplasia after endovascular injury (Tian et al., 2013). Here, PVAT from mice over-expressing Angptl2 accelerate neointima formation, while PVAT from Angptl2^−/−^ mice attenuated it. One should note, however, that the role of VSMC migration and proliferation in atherosclerosis is still debated (Bennett et al., 2016). Recent studies suggest that proliferation of VSMC is mainly protective, stabilizing the late-stage plaque rather than contributing to its formation (Bennett et al., 2016). Experiments in which PVAT has been replaced by other adipose tissue depots also hint to the similarities in paracrine signaling between PVAT and WAT. Two studies have examined the effects of replacing femoral PVAT with VAT and SAT, respectively, on neointimal formation (Takaoka et al., 2009; Tian et al., 2013). Interestingly, both studies arrived at the same result: removal of PVAT exacerbates neointimal hyperplasia, and transplantation of either VAT or SAT attenuates this effect (Takaoka et al., 2009; Tian et al., 2013). These studies corroborate previous reports that femoral PVAT is similar to WAT (Brown et al., 2014).

TNF-α and IL-6 are also known to accelerate atherogenesis, although evidence of PVAT directly affecting neointima formation via these molecules is lacking (Moe et al., 2013; Hartman and Frishman, 2014). The role of IL-6 seems to be dose-dependent: several studies have confirmed the pro-inflammatory effects of IL-6 in atherosclerosis models (Hartman and Frishman, 2014), but a complete loss of IL-6 increases plaque formation and serum cholesterol levels in ApoE^−/−^ mice (Schieffer et al., 2004).

Under homeostatic conditions, the anti-inflammatory effects of PVAT predominate, and secretion of pro-inflammatory paracrine agents is relatively low (Police et al., 2009; Fitzgibbons and Czech, 2014). In hypertriglyceridemia and obesity, however, there is significant upregulation of several pro-inflammatory chemokines and macrophage markers in both thoracic and abdominal PVAT (Police et al., 2009). The inflammatory response in thoracic PVAT, however, is quite low in comparison to abdominal PVAT, further supporting the notion that thoracic and abdominal PVAT closely resemble BAT and WAT, respectively (Police et al., 2009; Padilla et al., 2013).

The BAT-like phenotype of thoracic PVAT may also influence the progression of atherosclerosis in an inflammation-independent manner. Cold exposure activates both BAT and thoracic PVAT (Chang et al., 2012), initiating thermogenesis via mitochondrial uncoupling. The high requirement for fuel in this process leads to a dramatic increase in the uptake of circulating triglycerides into BAT, greatly reducing the levels of serum lipoprotein particles in both genetic and diet-induced models of obesity (Bartelt et al., 2011). In fact, activation of BAT has been shown to directly protect from atherogenesis through this mechanism in a recent study (Berbée et al., 2015). Interestingly, this study also addressed lipid uptake into PVAT during stimulation with the β3-AR agonist CL316243. Treatment with the agonist significantly increased uptake of lipids from the circulation in manner similar to BAT (Berbée et al., 2015), suggesting that cold exposure could also lead to an amelioration of diet-induced hypertriglyceridemia through PVAT activation, which in turn would slow the progression of atherosclerosis. Interestingly, activation of PVAT through cold exposure has been shown to reduce the expression of pro-inflammatory markers in ferrets, indicating a possible therapeutic role of PVAT in atherosclerosis and other inflammatory vascular diseases (Reynés et al., 2017).

The effects of aging also highlight the phenotypical differences between thoracic and abdominal PVAT. Padilla et al. investigated the expression of several inflammation-related genes in adipose tissues of young and old rats. Although neither thoracic nor abdominal PVAT showed any strong age-related increase in any of the analyzed inflammation markers, basal expression of almost all analyzed inflammatory markers was increased in abdominal compared to thoracic PVAT (Padilla et al., 2013). Moreover, abdominal, but not thoracic, PVAT of older rats had higher level of CD11α and FoxP3, indicating increased immune cell infiltration (Padilla et al., 2013). Bailey-Downs et al. also studied the combined effects of aging and HFD on inflammation in thoracic PVAT in mice (Bailey-Downs et al., 2013). Conditioned medium containing the secretome from PVAT excised from young or old mice fed either HFD or control diet (CD) was applied to aortic segments, and the inflammatory response of the vessels were analyzed. Interestingly, segments incubated with the conditioned medium from HFD-PVAT had higher levels of TNFα and IL-6 expression than segments incubated with conditioned medium from CD-PVAT (Bailey-Downs et al., 2013). Analysis of the PVAT itself after HFD or CD feeding did not reveal any significant increase in macrophage infiltration. This may indicate that while HFD does not necessarily lead to an increased state of inflammation in thoracic PVAT itself, it may contribute to inflammation in the vessel wall through paracrine signaling. In this study, aging strongly exacerbated the effects of HFD, both in terms of vessel inflammation and macrophage infiltration in PVAT (Bailey-Downs et al., 2013).

### PVAT and ROS production

Recently, a role for PVAT in regulating reactive oxygen species (ROS) production in vessels was described. ROS production by NADPH oxidases is a critical step for the development of endothelial dysfunction in several pathologies, including diabetes, atherosclerosis, and aging (Guzik et al., 2000; Guzik and Harrison, 2006). Analysis of the internal mammary arteries and the adjacent PVAT in a cohort of 386 patients revealed a strong correlation of insulin resistance and type 2 diabetes, with both lower serum adiponectin levels and increased ${\text{O}}_{2}^{-}$ production in the vascular wall. Additionally, artery segments incubated with adiponectin had significantly reduced vascular NADPH-dependent ${\text{O}}_{2}^{-}$ production, and scavenging ${\text{O}}_{2}^{-}$ radicals reduced adiponectin expression in PVAT. This study indicates that PVAT can detect ROS production in its adjacent artery, and in turn reduce it by increasing adiponectin expression (Antonopoulos et al., 2015).

ROS production in PVAT has also been implicated in obesity. Aortic PVAT from mice fed a HFD showed an increased TNF-α mediated ROS production, with increased contractile tone of the underlying vessel (Ketonen et al., 2010; da Costa et al., 2017). ROS production in thoracic PVAT following HFD is also known to be exacerbated by aging (Bailey-Downs et al., 2013). Whether or not this HFD-induced ROS production is partly responsible for the progression of e.g., atherosclerosis has not been directly studied.

### PVAT in hypertension

Hypertension is a common complication of obesity and affects almost 60% of obese individuals (Must et al., 1999). While the development of hypertension in obesity is multifactorial, it can partly be ascribed to the endocrine effects of adipose tissue (Sharma et al., 2001; Re, 2009). In obesity, WAT releases several factors that are known to affect vascular tone and blood pressure (Yiannikouris et al., 2010). These include leptin (Bravo et al., 2006), angiotensin II (Schütten et al., 2017), non-esterified fatty acids (NEFAs) (Sarafidis and Bakris, 2007), adiponectin (Ohashi et al., 2011), and resistin (Zhang et al., 2017). Interestingly, all these factors have been shown to be released from PVAT (see also above) (Lu et al., 2010; Gálvez-Prieto et al., 2012; Campia et al., 2014; Park et al., 2014; Antonopoulos et al., 2015). While this could indicate that PVAT is similar to WAT in terms of its regulation of hypertension, studies examining the role of BAT in hypertension are lacking. Furthermore, in some studies examining the release of the above mentioned factors, it is not immediately clear which type of PVAT was studied, making a thorough comparative analysis impossible (Gálvez-Prieto et al., 2012; Park et al., 2014). PVAT also releases numerous factors that directly affect vascular tone in a paracrine fashion, including norepinephrine, nitric oxide, hydrogen sulfide, and methyl palmitate (Lee et al., 2011; Chang et al., 2013). However, relating the release of these factors from PVAT to the effects of WAT on systemic vascular tone in obesity is not meaningful.

## Outlook

As the obesity pandemic continues to grow, there is an urgent need for effective pharmacological treatment of both the underlying causes and the associated comorbidities. Understanding the basic biology of the implicated tissues, as well as the pathological alterations and processes, is essential for the development of new therapeutic strategies. So far, the understanding of the role of PVAT in vascular disease is in its infancy, but recent studies indicate that several features of PVAT could have positive effects on the progression of atherosclerosis and endothelial dysfunction in various ways.

While the origin of PVAT seems to be different from BAT, their phenotypes appear almost indistinguishable, both in thermogenic properties and resistance to diet-induced inflammation. The thermogenic capacity of thoracic PVAT, for example, could prove useful in regulating energy expenditure, and should be investigated for a potential role in the treatment of energy balance-related diseases such as obesity and diabetes. Phenotypically and functionally, it is tempting to categorize thoracic PVAT as BAT, and abdominal and femoral PVAT as WAT. However, the developmental origins of PVAT seem to differ from both BAT and WAT, although this remains to be fully elucidated. In summary, the available evidence points to clear differences between PVAT and other adipose tissues. Thus, PVAT might be regarded as neither BAT nor WAT, but rather as a distinct type of adipose tissue.

## Author contributions

All authors listed have made a substantial, direct and intellectual contribution to the work, and approved it for publication.

### Conflict of interest statement

The authors declare that the research was conducted in the absence of any commercial or financial relationships that could be construed as a potential conflict of interest.
